# Differences in Inpatient Total Costs in Traumatic Brain Injury: A Retrospective Analysis from a Romanian Tertiary Care Center

**DOI:** 10.3390/healthcare13192466

**Published:** 2025-09-28

**Authors:** Iulia-Maria Vadan, Diana Grad, Stefan Strilciuc, Adina Stan, Vitalie Vacaras, Olivia Verisezan Rosu, Emanuel Stefanescu, Livia Livint-Popa, Alina Vasilica Blesneag, Dafin F. Muresanu

**Affiliations:** 1 Department of Neurosciences, “Iuliu Hațieganu” University of Medicine and Pharmacy, 400012 Cluj-Napoca, Romania; adinadora@yahoo.com (A.S.); vvacaras@umfcluj.ro (V.V.); livia.popa@umfcluj.ro (L.L.-P.); alinablesneag@yahoo.com (A.V.B.); dafinm@ssnn.ro (D.F.M.); 2 RoNeuro Institute for Neurological Research and Diagnostic, 400364 Cluj-Napoca, Romania; diana.grad@brainscience.ro (D.G.); stefan.strilciuc@brainscience.ro (S.S.); dr.olivia.rosu@gmail.com (O.V.R.); stefanescu.emanuel@yahoo.com (E.S.); 3 Department of Genomics, MEDFUTURE Institute for Biomedical Research, “Iuliu Hațieganu” University of Medicine and Pharmacy, 400012 Cluj-Napoca, Romania; 4 Neurology Clinic, Cluj County Emergency Clinical Hospital, 400347 Cluj-Napoca, Romania

**Keywords:** traumatic brain injury (TBI), healthcare costs, inpatient total hospital costs, Eastern Europe, cost comparisons, Romania, economic burden, clinical determinants, demographic factors, social determinants, post-traumatic amnesia

## Abstract

**Introduction:** Traumatic brain injury (TBI) represents one of the leading health concerns worldwide, and it is associated with high morbidity, mortality, and substantial healthcare costs. This study aimed to assess inpatient cost determinant factors among TBI patients admitted to an Eastern European hospital, Cluj County Emergency Hospital, Romania, in 2022. **Methods:** A retrospective observational analysis was conducted on 90 TBI patients. Data on demographic factors, clinical variables, injury characteristics, and inpatient hospital costs were collected. Inpatient total cost differences considering categorical variables were analyzed using Wilcoxon and Kruskal–Wallis tests, and correlations of inpatient total costs with other continuous variables were analyzed using Spearman correlations. **Results:** Most patients were male (67.8%), urban residents (67.8%), retired (64.4%), and had a mild TBI (96.7%), and the main listed cause was falls (74.4%). The average inpatient cost was EUR 1115.79. There were no statistically significant differences for costs in TBI severity, PTA, or comorbidities. Inpatient costs were correlated with hospital length of stay (ρ = 0.979, 95% CI rho: 0.969 and 986, *p* < 0.001). While higher costs were seen in patients with PTA, more comorbidities, severe Marshall Scores, or return-to-work status, these differences were not statistically significant. **Conclusions:** Further research with larger, multicenter cohorts is needed to better understand the cost structure of TBI care and to inform policy decisions that are aimed at resource allocation and cost efficiency.

## 1. Introduction

Traumatic brain injury (TBI) remains one of the most pressing healthcare problems worldwide, representing the leading cause of disability and death, especially in the group of people aged ≥65 years in developed countries [1].

According to the Global Burden of Disease study, there were 169,215 incident cases and 434,844 prevalent cases in Romania in 2016 [2,3,4].

It is estimated that their incidence will continue to rise and that during their lifetime, more than half of the world’s population will suffer a TBI—an alarming public health concern [3,5].

It is estimated that TBI represents a global burden of approximately USD 400 billion annually, and this is why healthcare professionals and policymakers worldwide are tracking the distribution of resources, treatment, and its associated costs in order to ensure high-quality care at the lowest cost possible [5,6]. It is expected that in the future, the incidence of TBIs will increase due to the increasing number in low- to middle-income countries of road traffic accidents and the growing elderly population in the majority of high-income countries [5,7,8]. Several articles highlight that the substantial global burden of TBIs necessitates prioritized efforts in prevention, optimization of clinical care, and advancement of research to reduce its impact on patients and healthcare systems [6,9,10,11].

Previous studies reported a TBI cost ranging from USD 2130 to USD 401,808 for severe cases and from USD 3079 to USD 7800 for mild ones. The higher the severity of the injuries, the higher the cost of the hospital stay [6,12]. Moreover, personnel costs and the implementation of clinical guidelines recommendations differ between countries and hospitals, resulting in different cost patterns [6]. However, differences in cost estimates are influenced by multiple factors, especially with regard to cost of illness studies: type of costs collected (direct medical, direct non-medical, and indirect costs), study perspective (societal, healthcare system, government, families, third-party payer, and business), prospective or retrospective, approaches employed in calculating costs (top-down, bottom-up, or econometric), as well as data availability and quality, as other research has evaluated, as well as sociodemographic or clinical predictors [13,14,15].

The Glasgow Coma Scale (GCS), the most commonly used score in clinical examination, helps categorize TBIs into mild, moderate, and severe. It is considered predictive of the patient’s long-term outcome [16]. However, some research emphasizes that the presence of post-traumatic amnesia (PTA) represents a greater predictor of costs than GCS in these patients [17]. The cause of injury also represents an important cost determinant, as it is known that in higher-income countries, falls represent a main cause of injury, while in lower- to middle-income countries, road traffic accidents are the majority cause [5,9,18,19]. These need and utilize higher resources, thus increasing the cost of treatment. A history of drug use and intoxication at admission was also linked to longer hospital stays, higher mortality, and poorer discharge outcomes [20,21,22,23]. The level of education was linked to cognitive rehabilitation following an injury [24] and to a possible earlier return to work [25]. There are also studies that evaluate the marriage stability of individuals after a TBI or how it may affect the rehabilitation of a patient [26,27,28]. The place of residence (urban vs. rural) can influence costs due to access to healthcare services [29]. Patients with more severe injuries and a higher Modified Marshall Score need more complex treatment, thus increasing resource utilization [30] and resulting in higher costs of care. In addition, the return-to-work rate is important because it signifies the successful treatment and rehabilitation of a patient [25,28,31,32]. Collectively, these factors constitute key determinants of the overall costs associated with the treatment and rehabilitation of patients with TBIs.

To our knowledge, there are no studies aiming to analyze group differences in inpatient total costs in Romania or correlations with inpatient total costs, which makes this study the first one to address a critical gap in the literature by providing a comprehensive analysis of inpatient total costs group differences considering different types of variables associated with TBI in Romania. This study provides context-specific evidence to inform health policy by investigating the differences between clinical, demographic, and social determinants and inpatient outcomes.

## 2. Methods

### 2.1. Study Design

This is a single-center, retrospective, observational study, analyzing inpatient total costs for patients diagnosed with a TBI who were admitted to the Cluj County Emergency Hospital in 2022 (between 9 January and 12 December 2022).

### 2.2. Variables

We analyzed the following categorical variables: alcohol consumption (categories: no use, abuse, regular use, unknown); medical care level (neurology, neurosurgery—both inpatient); cause of injury (fall, road traffic accident, aggression); comorbidity burden (0, 1, >1); discharge location (home, other, another hospital, rehabilitation); discharge status (dead, alive); drug use (yes, no); education (primary school (1–4 years), secondary school (5–8 years), high school (9–12 years), university (13+ years), no formal education, unknown); employment (retired, employed, unemployed, other, unknown); GCS severity (mild, moderate, severe); imagery (yes, not); imagery info (CT, MRI); initial referral (ICU, neurology and neurosurgery—inpatient); marital status (married, living together, widowed, single, separated, unknown); mechanism of injury (ground-level fall, direct impact; fall from height >1 m (3 ft), acceleration/deceleration, direct impact: blow to the head, crush, and missing data); Modified Marshall score (I, II, III, IV, V); place of injury (home, street, other, sports facility); post traumatic amnesia (no, yes); return to work (yes, no, unknown); settlement type (rural, urban); sex (male female); smoking (no use, regular use, abuse); substance use (no use, regular use, abuse); type of injury (closed, crush, piercing).

For continuous data, we analyzed the following variables: age, length of stay, ISS score, and inpatient total cost, which was collected for each hospitalization. We converted the cost from RON to EUR, based on an average for 2022 retrieved from the European Central Bank [33].

The dependent variable was the inpatient total cost.

### 2.3. Statistical Analysis

We report descriptive statistics based on the type of the variables: count and percentage for categorical variables; and mean and median for continuous variables.

We used the Shapiro–Wilk test to assess the normality of the data for inpatient total costs, hospital length of stay, age, and ISS, and based on the results (inpatient total costs: W = 0.933, *p*-value = 0.0001; hospital length of stay: W = 0.948, *p*-value = 0.0014; age: W = 0.928, *p*-value = 0.0001; and ISS score: W = 0.821, *p*-value < 0.001), the data was not normally distributed. These findings were sustained by visual inspection as well. The data maintained its deviation from a normal distribution even after log transformation; therefore, we decided to proceed with the original, non-transformed data. We used non-parametric tests, such as the Wilcoxon rank-sum test (Mann–Whitney U), the Kruskal–Wallis rank-sum test, and Spearman’s correlation.

We performed the Wilcoxon test for cost differences among categorical variables (sex, settlement type, post-traumatic amnesia, discharge status) with only two categories, and the Kruskal–Wallis test for variables with more than two categories (cause of injury, number of comorbidities, return to work, smoking, and Modified Marshall score). We performed Spearman correlations for inpatient total costs and length of stay, inpatient total costs and ISS score, and inpatient total costs and age. The strength of the correlation was assessed based on the value of Spearman’s correlation coefficient—rho, which ranges from −1 to 1. The strength of the correlation was categorized as follows: “0.0 < 0.1—no correlation, 0.1 < 0.3—low correlation, 0.3 < 0.5—medium correlation, 0.5 < 0.7—high correlation, and 0.7 < 1 very high correlation” [34].

The alpha level was set at *p* < 0.05. All analyses were conducted in R v4.3.3.

## 3. Results

### 3.1. Descriptive Statistics

Our sample consisted of 90 patients. Most of them were male, living in urban areas (67.8%), had more than one comorbidity (42.2%), listed university as the highest level of education achieved, were retired (64.4%), and were married (52.2%). Most did not smoke (74.4%) or consume alcohol (60%). The primary cause of injury was falls (74.4%). The mechanism of injury was predominantly a ground-level fall (65.6%) and home was listed as the place of injury (66.7%) for most patients. Based on admission GCS, most patients were diagnosed with a mild TBI (96.7%). Post-traumatic amnesia was present in 21.1% of TBI patients. Only 5.6% died, while 93.3% were discharged home and 51.1% returned to work. Additional details can be found in Table 1.

**Table 1:** Descriptive statistics for 90 TBI patients.

		n	%
Alcohol consumption	No use	54	60.0%
	Abuse	20	22.2%
	Regular use	15	16.7%
	Unknown	1	1.1%
Cause of injury	Fall	67	74.4%
	Road traffic accident	21	23.3%
	Aggression	2	2.2%
Comorbidity burden	>1	38	42.2%
	1	28	31.1%
	0	24	26.7%
Discharge location	Home	84	93.3%
	Other	5	5.6%
	Rehabilitation	1	1.1%
Discharge status	Alive	85	94.4%
	Dead	5	5.6%
Drug use	No	90	100.0%
Education	University (13+ years)	58	64.4%
	High school (9–12 years)	25	27.8%
	Primary school (1–4 years)	3	3.3%
	No formal education	2	2.2%
	Secondary school (5–8 years)	1	1.1%
	Unknown	1	1.1%
Employment	Retired	58	64.4%
	Employed	22	24.4%
	Unemployed	8	8.9%
	Other	1	1.1%
	Unknown	1	1.1%
GCS severity	Mild	87	96.7%
	Moderate	3	3.3%
Imagery	Yes	90	100.0%
Imagery information	CT	90	100.0%
Marital status	Married	47	52.2%
	Widowed	19	21.1%
	Single	18	20.0%
	Separated	3	3.3%
	Unknown	2	2.2%
	Living together	1	1.1%
Mechanism of injury	Ground-level fall	59	65.6%
	Direct impact: head against an object	12	13.3%
	Fall from height >1 m (3 ft)	10	11.1%
	Acceleration/deceleration	4	4.4%
	Direct impact: blow to the head	3	3.3%
	Crush	1	1.1%
	Missing data	1	1.1%
Modified Marshall score	II	80	88.9%
	I	7	7.8%
	III	3	3.3%
Place of injury	Home	60	66.7%
	Street	27	30.0%
	Other	2	2.2%
	Sports facility	1	1.1%
Post traumatic amnesia	No	71	78.9%
	Yes	19	21.1%
Return to work	Yes	46	51.1%
	No	23	25.6%
	Unknown	21	23.3%
Settlement type	Urban	61	67.8%
	Rural	29	32.2%
Sex	male	61	67.8%
	female	29	32.2%
Smoking	No use	67	74.4%
	Regular use	21	23.3%
	Abuse	2	2.2%
Substance use	No use	90	100.0%
Type of injury	Closed	88	97.8%
	Crush	1	1.1%
	Penetrating	1	1.1%

**Figure 1:**
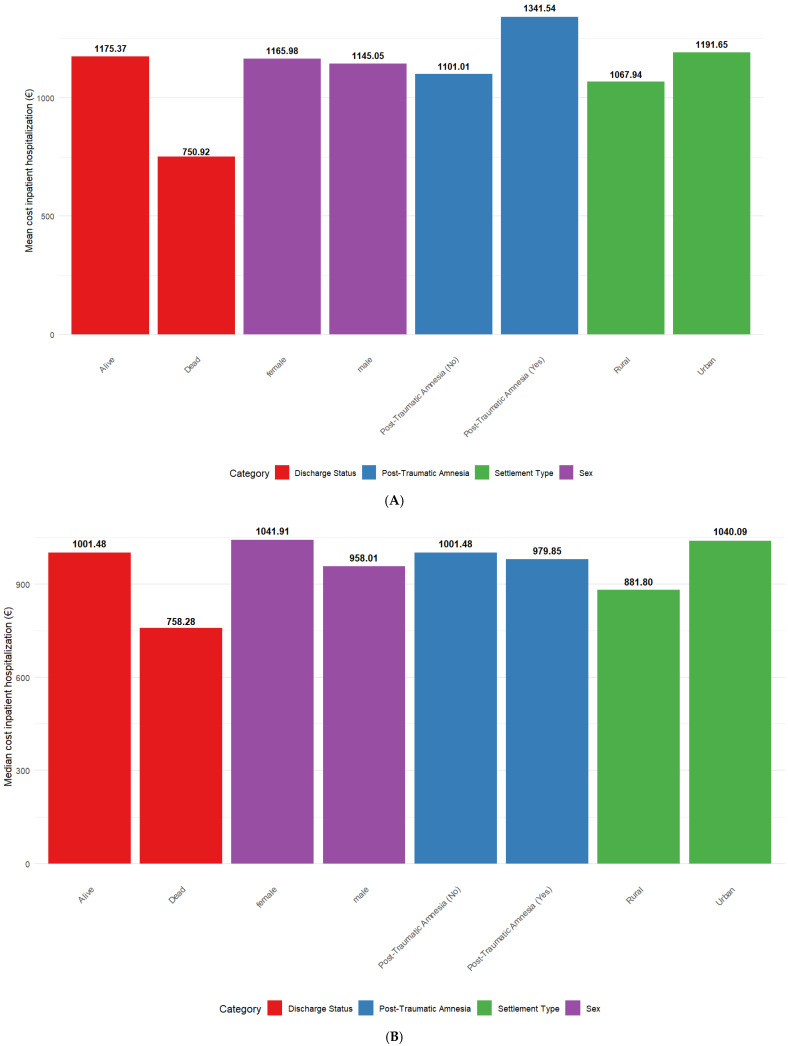
(**A**,**B**) Mean and median costs for inpatient hospitalizations for TBI patients stratified by discharge status, post-traumatic amnesia, sex, and settlement type.

**Figure 2:**
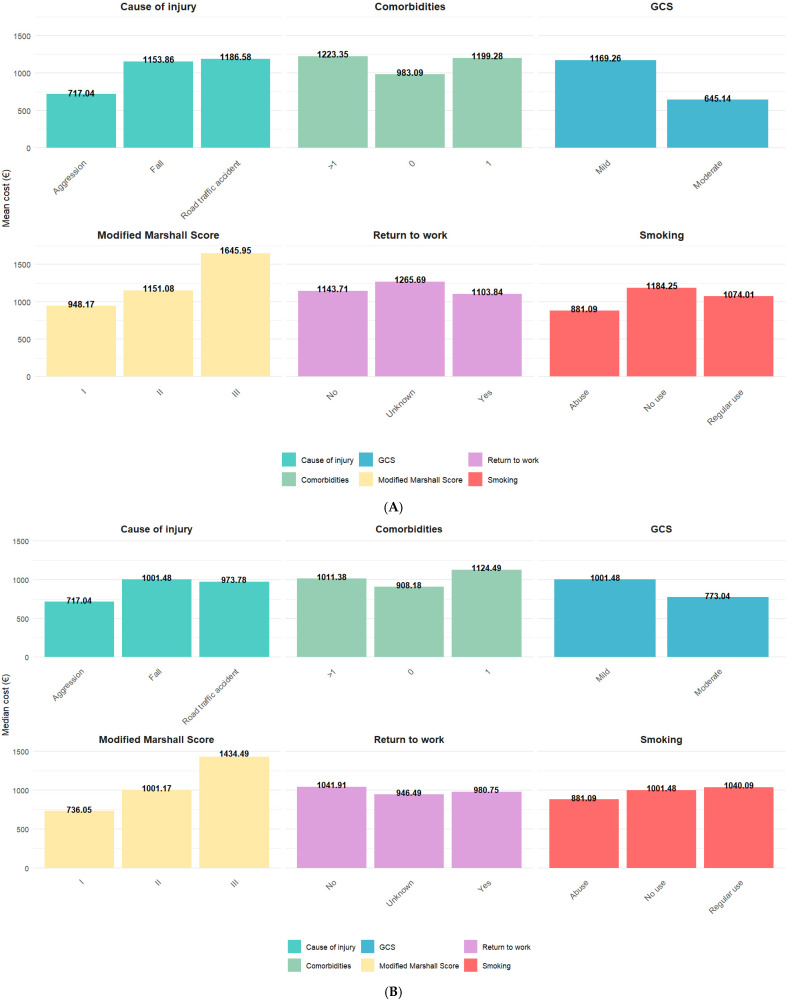
(**A**,**B**) Mean and median costs for inpatient hospitalizations for TBI patients stratified by cause of injury, number of comorbidities, GCS, Modified Marshall Score, return to work, and smoking.

The mean was 64.8 (median: 69.5) for age, 7 days (median 6.5) for length of stay, EUR 1115.79 (median: EUR 1001.17) inpatient hospitalization cost for the entire sample, and 5.62 (median: 3) for ISS. In Figure 1, the highest mean costs were registered for TBI patients with post-traumatic amnesia (EUR 1341.54) while the lowest were registered for TBI patients that died (EUR 750.92). While the lowest median costs were registered among TBI patients that died (EUR 758.28), the highest median costs were among female TBI patients (EUR 1041.91). The highest variation within categories was found for discharge status for both mean (alive—EUR 1175.37; dead—EUR 750.92) and median (alive—EUR 1001.48; dead—EUR 758.28). In Figure 2, the highest mean costs were registered for TBI patients with a Modified Marshall Score—III (EUR 1645.95) while the lowest were registered for TBI patients with moderate a TBI (EUR 645.14). While the highest median costs were registered among TBI patients with a Modified Marshall Score—III (EUR 1434.49), the lowest median costs were among TBI patients that listed aggression as the cause of injury (EUR 717.04). The highest variation within categories was found for the Modified Marshall Score for both mean and median.

### 3.2. Wilcoxon Test

When comparing the costs between: (a) male and females (W = 932, *p*-value = 0.684, difference in location: 50.94, 95% CI for the difference in location: −250.26 and 293.12); (b) settlement types—rural and urban (W = 754, *p*-value = 0.261, difference in location: −126.75, 95% CI for the difference in location: −366.91 and 125.74); (c) those with and without post traumatic amnesia (W = 595, *p*-value = 0.434, difference in location: −138.07, 95% CI for the difference in location: −503.70 and 16.91); (d) patients that were discharged dead or alive (W = 283, *p*-value = 0.217, difference in location: 373.70, 95% CI for the difference in location: −214.08 and 965.49); and (e) patients of mild and moderate TBI severity (w = 78, *p*-value = 0.242, difference in location: −404.51, 95% CI for the difference in location: −1272.96 and 278.16), the differences were not statistically significant.

### 3.3. Kruskal–Wallis

Based on the results of the Kruskal–Wallis rank-sum tests to evaluate potential differences in inpatient costs between the categories of the following variables: cause of injury (χ^2^ = 1.015, *p* = 0.602), Modified Marshall score (χ^2^ = 3.9303, *p* = 0.140), number of comorbidities (χ^2^ =2.613, *p* = 0.270), return to work (χ^2^ = 0.8266, *p* = 0.661), and smoking (χ^2^ = 0.7329, *p* = 0.693), no statistically significant results were found.

### 3.4. Spearman Correlations

Between inpatient costs and hospital length of stay, there was a very high positive correlation (rho = 0.979, 95% CI rho: 0.969 and 0.986, *p*-value < 0.001), while between inpatient costs and age, there was a low positive, non-statistically significant correlation (rho = 0.121, 95% CI rho: −0.087 and 0.320, *p*-value = 0.253). As for inpatient total costs and ISS, there was no correlation (rho = 0.060, 95% CI rho: −0.148 and 0.264, *p*-value = 0.569).

## 4. Discussion

This retrospective observational study aimed to analyze group differences and correlations of inpatient total costs for TBIs at Cluj County Emergency Hospital, an Eastern European tertiary care center, in 2022. A total of 90 patients were included in the study, with the majority being admitted to the neurology ward. The predominant gender affected was male (67.8%), which is similar to other reports in the literature [6,35,36]. Most injuries occurred at home to people living in urban areas (67.8%) and who were retired (64.4%), the latter being consistent with other articles that indicate that the elderly are more inclined to suffer from a TBI [17,37]. Regarding the costs of treatment, Hu et al. [35] found that the median cost was lower for individuals aged 65 years or older than for those aged 18–64, possibly due to the severity of the TBI and the length of hospital stay. The results of the current paper appear to be in contrast with some studies that have found age to be a factor in the complexity and cost of care for TBI patients [38,39], suggesting that the relationship between age and healthcare costs may depend on the population and healthcare system studied.

Additionally, as shown in Figure 1A,B, female patients had slightly higher mean (EUR 1165.98 vs. EUR 1145.05) and median (EUR 1041.91 vs. EUR 958.01) hospitalization costs than male patients. However, no statistically significant differences were found between males and females based on the performed non-parametric test. This finding is supported by some studies [40] and contradicted by others [6]. Differences in injury severity, the presence or absence of comorbidities, or health-seeking behaviors might have influenced the results, although further investigation is needed to clarify these patterns.

Regarding the rural versus urban setting, patients residing in urban areas had higher hospitalization costs than those from rural areas, both in terms of mean (EUR 1191.65 vs. EUR 1067.94) and median (EUR 1040.09 vs. EUR 881.80) values. However, no statistically significant differences were found between rural and urban areas based on the performed non-parametric test (e.g., Wilcoxon rank-sum). Similar articles in the literature show that a higher number of TBIs occur in urban areas, but the number of injury-related deaths is higher in rural areas [41], possibly due to faster access to care in urban areas, while a lack of resources in rural areas results in poorer results [42].

Figure 1 also shows that patients who survived their hospitalization had higher costs than those who died, with mean costs of EUR 1175.37 versus EUR 750.92, and median costs of EUR 1001.48 versus EUR 758.28, respectively. However, no statistically significant differences were found between survivors and non-survivors based on the performed non-parametric test. There are other studies from the literature that highlight that surviving TBI patients impose a substantial long-term financial burden [43,44]. This difference may be due to a greater need for medical resources and a longer duration of care for patients who eventually recover, compared to those with fatal outcomes whose hospitalizations were shorter and required fewer resources, resulting in a lower final cost compared to the others. In contrast, some studies indicate that the acute care for patients who do not survive hospitalization is more expensive than that for those who recover [45].

The most common cause of injury was falling (74.4%), especially ground-level falls (65.6%) that occurred at home, emphasizing the public health importance of fall prevention among the elderly, who made up a significant portion of our sample, a finding also supported by other studies [46,47,48]. However, there were no statistically significant group differences for inpatient total costs for either the cause or the mechanism of TBIs. Figure 2A,B reveal that TBIs resulting from aggression had the lowest mean and median costs (EUR 717.04), whereas falls and road traffic accidents had higher costs (mean: EUR 1153.86 and EUR 1186.58; median: EUR 1001.48 and EUR 973.78, respectively), suggesting that falls and traffic-related trauma often result in more severe or multifocal injuries requiring extended care and diagnostics, a fact that has also been found in other research articles [44,49,50,51]. However, due to the small sample size and the nature of the data, the assumptions required to perform different types of regressions were not met, making it impossible to investigate this further. Moreover, a smaller number of patients (23.3%) were involved in road traffic accidents in this sample.

A very high positive correlation was found between inpatient costs and length of stay (Spearman’s rho = 0.979, *p* < 0.001), underlining that longer hospitalizations are correlated with increased costs. This finding is consistent with the existing literature, where extended hospital stays, often due to complications or slower recovery, are correlated with higher healthcare expenses [38,52,53,54].

The current research did not find any statistically significant group differences between inpatient costs and clinical severity measures such as GCS classification, the Modified Marshall Score, or correlation with the ISS score. Considering Figure 2, the highest average costs were observed in mild TBI patients (mean: EUR 1169.26; median: EUR 1001.48), whereas moderate TBI patients had the lowest average costs (mean: EUR 645.14; median: EUR 773.04), a result that seems to be in opposition to other research [6], most likely due to the imbalance in patient severity (only 3 moderate TBI patients vs. 87 mild ones). In regard to the Modified Marshall Score, used to classify structural brain injury severity, patients with Score III had the highest mean (EUR 1645.95) and median (EUR 1434.49) hospitalization costs, emphasizing the economic burden of severe brain injuries and the complexity of care needed by these patients in acute settings. However, no statistically significant group differences for inpatient total costs were found between GCS categories, as well as the Modified Marshall score, based on the results of the performed non-parametric test. Additionally, the correlation between inpatient total costs and ISS was not statistically significant.

The presence of PTA was another factor analyzed in this study. Those with PTA had higher mean inpatient costs (EUR 1341.54 for PTA vs. EUR 1101.01 for no PTA), suggesting that the presence of it may be an indicator of more severe injury and prolonged recovery. The difference was not statistically significant, unlike other articles [17,55,56], where the presence of PTA was found to have a higher importance in determining the costs of treatment for patients. This may be due to the relatively small number of patients with PTA (21.1%), which limits the power of the statistical tests and suggests that it could potentially be an indicator of a more complex or prolonged need for care, as suggested by other articles that focused on rehabilitation for patients suffering from PTA [56,57,58]. Other studies have explored the influence of PTA on cost in TBI patients and showed that each additional day of PTA resulted in a higher total cost [55,56]. Further research with larger sample sizes is necessary to clarify this.

Regarding comorbidities, alcohol consumption, and smoking, even though there are not many studies that show how smoking influences the cost of TBI treatment, it is a known risk factor for other health complications, and its presence might increase the complexity of procedures needed and prolong the recovery period [59,60]. In the current paper, smokers and non-smokers exhibited similar costs, further indicating that other factors may contribute more significantly to the cost burden of TBIs. For comorbidities, the current literature implies that primarily, known psychiatric illness and treatment increase the costs of TBI treatment and recovery [17,61]. In this study, patients with no comorbidities had lower average costs (mean: EUR 983.09; median: EUR 908.18), while those with multiple comorbidities had higher costs (mean: EUR 1223.35; median: EUR 1011.38), possibly because patients with multiple comorbidities may have a more complicated recovery, leading to a more expensive treatment course, but the results were not statistically significant.

For the return-to-work status, as shown in Figure 2, there were slight differences in both mean and median inpatient total costs. Patients with an unknown return-to-work status had the highest mean cost (EUR 1265.69), followed by those who did not return to work (EUR 1143.71). Patients who successfully returned to work had lower costs (mean: EUR 1103.84; median: EUR 980.75), suggesting that more favorable functional outcomes may correspond to shorter or less resource-consuming hospitalizations. However, no statistically significant group differences for inpatient total costs were found among the different levels of this variable, based on the performed non-parametric test. Other studies in the literature suggest that returning to work implies lower long-term costs in patients [62,63]. Half of the patients in the study returned to work within 12 months post-TBI, compared to other studies where the percentage was higher [31,64].

### Study Strengths and Limitations

The current research has several strengths. It analyzes the cost of TBIs in an Eastern European hospital, providing valuable data from a region often underrepresented in the literature, by taking into account a wide range of variables—demographic, clinical, and economic. By converting the costs to euros using a standardized exchange rate, it facilitates the international comparability of the economic data. Additionally, by utilizing hospital data, it enhances the relevance of the findings, particularly in providing a valuable resource for health policymakers.

On the other hand, the study has several limitations. It is a single-center study, and as a result, the generalizability of the findings is limited. The sample size of patients was relatively small (n = 90), which can reduce the statistical power of the analysis. Additionally, several variables contain very small subgroups, which substantially increases the risk of Type II errors and may result in clinically meaningful associations failing to reach statistical significance. Moreover, our sample consisted mostly of patients with mild TBIs, with severe patients being completely underrepresented. The study focused solely on short-term inpatient costs and did not examine long-term outcomes such as rehabilitation costs, functional recovery, or quality of life. In addition, we analyzed only total costs, rather than cost categories, as the database used did not collect disaggregated types of costs. Most importantly, only total inpatient costs were available; data on specific cost categories and their individual contributions to total costs were not captured. Future research studies should encompass different cost categories, collected from multiple centers and from patients and caregivers as well, and should be combined with follow-up measurements on quality of life, in order to fully understand the multifaceted economic burden associated with TBIs.

## 5. Conclusions

The current research provides the first study on cost differences for several variables in patients with TBIs in Romania, filling a critical evidence gap in Eastern Europe. Our results highlight that the only statistically significant results were correlations between inpatient total costs and length of hospital stay. As for other variables (clinical, social, and demographic), there were no statistically significant group differences in inpatient total costs among different levels of the analyzed variables.

The predominance of falls as a major cause of injury in the elderly emphasizes the changing epidemiology of TBIs, consistent with trends in high-income countries. This marks the importance of preventive strategies, especially fall-prevention programs for older adults.

Despite its limitations, this study offers valuable insights into Romania regarding inpatient total costs across different variables in patients with TBIs and sets the foundation for future broader research. This research provides valuable evidence to help improve healthcare policies, optimize healthcare delivery, and enhance the efficiency and quality of TBI care in Eastern Europe.

In conclusion, optimizing hospital stays and expanding preventive and rehabilitation strategies should be the main focus to improve TBI care in this region. Future multicenter studies are essential in order to fully capture the economic burden of TBIs in Romania and Eastern Europe.

## Data Availability

Data available on request from the corresponding author due to the sensitive nature of the data.
